# EnsembleSplice: ensemble deep learning model for splice site prediction

**DOI:** 10.1186/s12859-022-04971-w

**Published:** 2022-10-06

**Authors:** Victor Akpokiro, Trevor Martin, Oluwatosin Oluwadare

**Affiliations:** 1 Department of Computer Science, University of Colorado, Colorado Springs, CO 80918 USA; 2 Department of Mathematics, Oberlin College, Oberlin, OH 44074 USA

**Keywords:** Splice sites (SS), Ensemble learning, Deep learning (DL), Convolutional neural network (CNN), Dense neural network (DNN), Feature extraction

## Abstract

**Background:**

Identifying splice site regions is an important step in the genomic DNA sequencing pipelines of biomedical and pharmaceutical research. Within this research purview, efficient and accurate splice site detection is highly desirable, and a variety of computational models have been developed toward this end. Neural network architectures have recently been shown to outperform classical machine learning approaches for the task of splice site prediction. Despite these advances, there is still considerable potential for improvement, especially regarding model prediction accuracy, and error rate.

**Results:**

Given these deficits, we propose EnsembleSplice, an ensemble learning architecture made up of four (4) distinct convolutional neural networks (CNN) model architecture combination that outperform existing splice site detection methods in the experimental evaluation metrics considered including the accuracies and error rates. We trained and tested a variety of ensembles made up of CNNs and DNNs using the five-fold cross-validation method to identify the model that performed the best across the evaluation and diversity metrics. As a result, we developed our diverse and highly effective splice site (SS) detection model, which we evaluated using two (2) genomic *Homo sapiens* datasets and the *Arabidopsis thaliana* dataset. The results showed that for of the *Homo sapiens* EnsembleSplice achieved accuracies of 94.16% for one of the acceptor splice sites and 95.97% for donor splice sites, with an error rate for the same *Homo sapiens* dataset, 4.03% for the donor splice sites and 5.84% for the *a*cceptor splice sites datasets.

**Conclusions:**

Our five-fold cross validation ensured the prediction accuracy of our models are consistent. For reproducibility, all the datasets used, models generated, and results in our work are publicly available in our GitHub repository here: https://github.com/OluwadareLab/EnsembleSplice

## Background

**Fig. 1:**
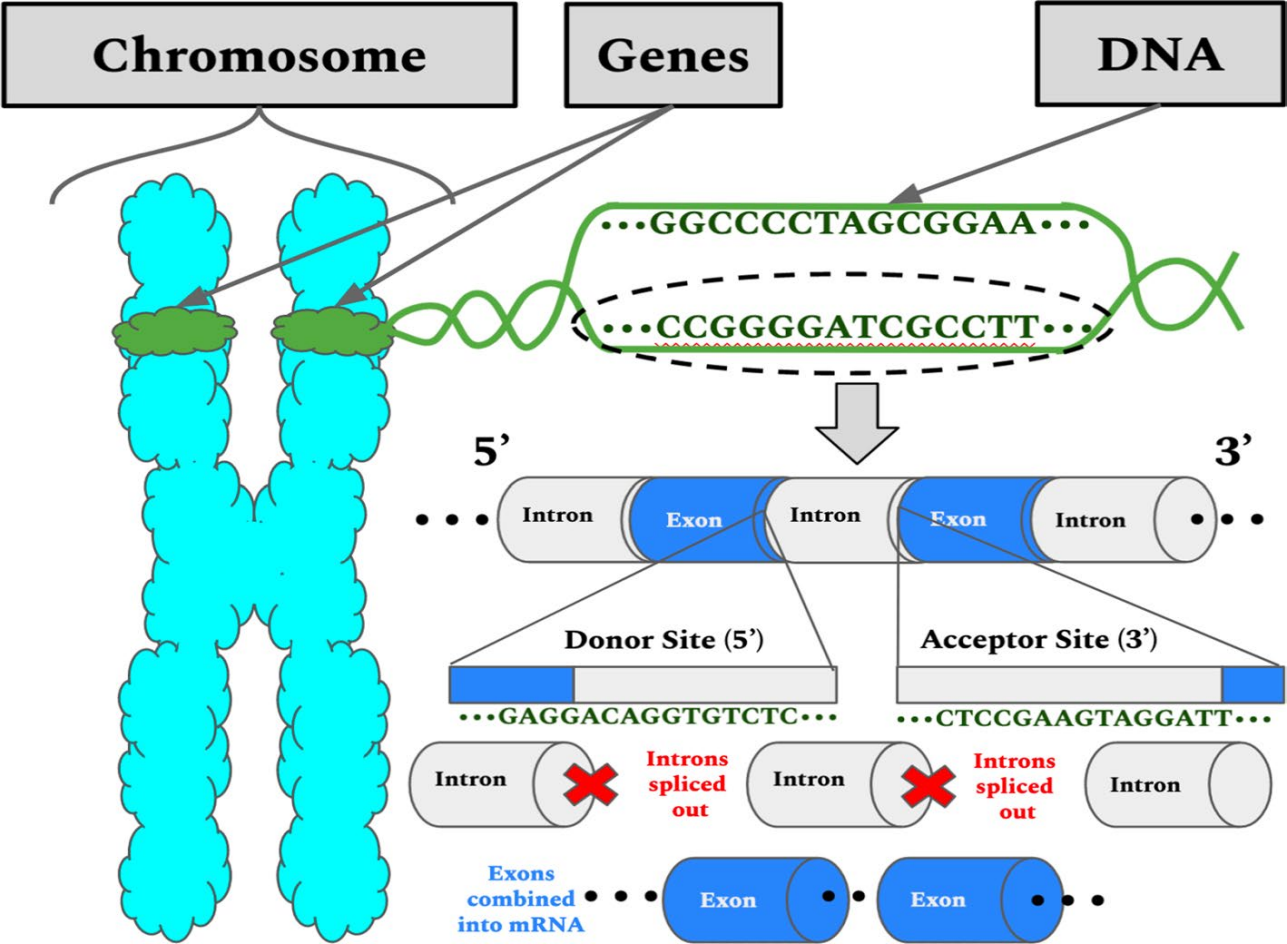
Illustration of 2 step biochemistry process for Splice Sites. This figure shows canonical sequence distribution in a splice site location, the Introns are spliced, hence the name splice sites resulting in proteins as a final product

The development of high-throughput computational sequencing methods and technologies has created a significant opportunity for gene structure analysis research and experiments. We focus on splice sites detection in this paper, which is critical for gene structure and expression analysis. The gene sequences essential for protein synthesis are composed of alternating nucleotide regions called introns, which are the non-protein-coding regions, and exons, which are the protein-coding regions. During DNA transcription in eukaryotic cells, an enzyme called spliceosomes cuts out introns and concatenates exons; this process is known as RNA splicing and is required for the creation of mature mRNA from pre-mRNA, which is required for gene expression and protein synthesis [1]. The dinucleotides AG and GT are biological markers involved in RNA splicing and are often found in the 3′ intron boundary, or donor splice site (DoSS) region, and the 5′ intron boundary, or acceptor splice site (AcSS) region, respectively [2] as shown in Fig. 1.

Organismal genomes are studied primarily through genome annotation, which involves classifying genomic elements based on their function and location [3]. This annotation is typically performed at the nucleotide level to determine the locations of key genetic elements in DNA sequences, as well as at the protein level to assess proteomic function and investigate the mechanisms underlying gene interaction and splice site localization [4]. More specifically, different computational methods have been proposed to accurately detect splice sites location, which can be used to identify genes in eukaryotic genomes. This biological and biochemical process has proven to be time-consuming and ineffective in the real world, necessitating the development of computational tools for accurate splice site prediction.

The earliest research on genomic DNA splice site prediction primarily leveraged methods in machine learning and probabilistic modeling. GeneSplicer was the first to achieve record accuracies with its Markov model-enhanced maximal dependence decomposition decision trees, which contributed to the popularity of Markov models for splice site prediction [2]. Other earlier works used Markov Model as a preprocessing technique for other algorithms such as shallow neural networks, or to enhance performance [5, 6]. Burge et al. [7] developed the MDD method [8] as a decision tree approach to reduce the computational burden of increasing the Markov model order. Goel et al. [9] proposed a method also based on Markov model. Some other methods adopted the use of support vector machines (SVMs) for their simplicity and speed [10, 11]. While the intricacy of these machine learning models grew, their accuracy plateaued. This was due to both compute power and the bottleneck of having to select the model's features manually.

Deep learning, along with better computing methods and resources, has largely solved these issues. In recent years, splice site prediction has been performed using the deep learning (DL) approach with neural networks (NNs). Convolutional Neural Networks (CNN) are the most frequent neural network (NN) architecture adopted for this deep learning approaches, and widely deviates in their depth (number of layers) and parameters across studies. SpliceRover [12], SpliceFinder [13], DeepSplicer [14], DeepSS [15], Spliceator [16], and iSS-CNN [17], among others, employ CNNs. Donor and acceptor sites are typically one-hot-encoded and batch-fed into these architectures, which perform feature extraction and exceed the earlier ML techniques in classification accuracy. On genomic DNA, other deep learning methods have been used, including the Long-Short Term Memory (LSTM) neural network and the Recurrent Neural Network (RNN), which are sequence learning networks commonly used in time-series analyses. SpliceViNCI, for example, is a bidirectional LSTM with integrated gradients [18].

In this work, we propose a stacking ensemble method for splice site prediction to combine various classifiers to produce an alpha-classifier that is more effective at classification and generalization than the individual classifiers. Through training, a stack (ensemble) of various neural networks models (base-models) develops its own representation of the genomic data. Following this, each network predicts the unidentified splice sequences on its own. These predictions are combined into a new dataset's pool entries. For example, if the ensemble included three different CNNs and two different DNNs, and the predictions for a splice site were [1], [1], [1], [0], [1] for each network, then the row of entries would read [1, 1, 1, 0, 1]. Following the creation of this new dataset, a final prediction using the new dataset is then made using simple logistic regression (meta-model). The main importance of ensemble learning is that the diversity of predictions balances out the weaknesses of individual base model performances, increasing overall accuracy and resulting in improved performance and robustness. This performance and robustness importance can be seen in other deep learning works of literature, including models for positioning footballers [19] in sport science research, models for predicting generic Escherichia coli population in agricultural ponds based on weather station measurements [20], and improving model performances for the detection of Alzheimer's disease [21] in health science research.

Our method combines deep neural network architectures to create EnsembleSplice, a novel ensemble architecture. Hence, we propose a deep learning architecture that learns from an ensemble of CNNs to achieve a state-of-the-art performance in true and false splice sites prediction accuracy and efficiency. We used grid search methods to determine the best hyperparameters, and the best ensemble selection was achieved using five-fold cross-validation, as shown in the manuscript's tables and results. Furthermore, we compare EnsembleSplice’s splice site identification performance to that of existing splice site tools using three genomic DNA datasets as benchmarks. The datasets, datasets preprocessing using one-hot encoding, EnsembleSplice pipeline, performance benchmarks methods, subsections are discussed in the methodology section, while explanatory evaluation metrics, cross-validation, result discussion and model interpretability subsections are discussed in the experiments and results section, as well as the conclusion sections.

In summary, the aim and objective of this work is as follow:

- Develop a deep ensemble model architecture consisting of DNNs and/or CNNs that achieves excellent performance on the task of splice site classification.
- Ensure via cross validation, that the deep ensemble consists of effective component neural networks (CNNs and/or DNNs) with high diversity across them.
- Ensure that our deep ensemble architecture is robust, with a minimum dispersion and consistent in performance in splice site prediction across different datasets, than current state-of-the-art algorithms.

## Methods

### Datasets

Each dataset used in this research consists of both confirmed true (positive) AcSS/DoSS and confirmed false (negative) AcSS/DoSS. Evaluation of classification performance is partitioned by splice site type. This means that EnsembleSplice is trained to distinguish between true and false DoSS regions and is trained again and separately to distinguish between true and false AcSS regions.

#### HS3D

The *Homo Sapiens* Splice Sites Dataset (*HS3D*) is a collection of human genomic DNA introns and exons extracted from GenBank Rel.123 [22] *HS3D'*s Primate Division. There are 2796 confirmed true DoSS regions, 2880 true positive AcSS regions, 271,937 confirmed false DoSS regions, and 329,374 confirmed false AcSS regions. This paper randomly selects 2750 false DoSS regions and 2750 false AcSS regions from the 271,937 and 329,374 available in the dataset, respectively; the Python code snippet *random.seed(123,454)* is used to shuffle the entire *HS3D* dataset before the false DoSS and false AcSS subsets are selected. The full set of 2750 confirmed true DoSS regions and 2750 confirmed true positive AcSS regions are used. The nucleotide consensus AG for AcSS regions occurs at positions 69 and 70, and the nucleotide consensus GT for DoSS regions occurs at positions 71 and 72. In total, each *HS3D* donor and acceptor site splice sequence is 140 nucleotides long, with this sequence length used for the cross-validation, performance, and comparison experiment. The *HS3D* dataset can be accessed at http://www.sci.unisannio.it/docenti/rampone/.

#### Homo sapiens and Arabidopsis thaliana

The *Homo sapiens* and *Arabidopsis thaliana* datasets consist of splice site regions selected from annotated genomic DNA sequences for Homo sapiens and *A. thaliana* in Ensembl 2018 [23]. Using Bedtools [24, 25], the peripheral nucleotide sequences padding each AcSS, or DoSS were determined. Each splice site region in these datasets is 602 nucleotides long; each DoSS region has consensus GT at positions 301 and 302, and each AcSS has consensus AG also at positions 301 and 302. There are 250,400 confirmed true and false DoSS regions and 248,150 confirmed true and false AcSS regions in the *Homo sapiens* dataset. There are 110,314 confirmed true and false DoSS regions and 112,336 confirmed true and false AcSS regions in the *A. thaliana* dataset. The confirmed true AcSS and DoSS regions were selected from chromosomes 21, 2, 2L, 1, and I. This paper randomly selects 8000 true and false DoSS regions (totaling 16,000 entries) and 8000 true and false AcSS regions (totaling 16,000 entries) from both datasets. As with the *HS3D* dataset, the Python code snippet *random.seed*(123,454) is used for shuffling the Homo sapiens and *A. thaliana* datasets before the DoSS and AcSS subsets are selected. The *Homo sapiens* and *A. thaliana* datasets can be accessed at https://github.com/SomayahAlbaradei/Splice_Deep.

We used the source sequence length—140 nucleotides for HS3D and 602 for *Homo sapiens* and *A. thaliana* datasets —as discussed in the subsections for all cross-validation, performance, and comparison experiments executed and results reported.

### One-hot encoding and hyper-parameter search space and tuning

Genomic DNA splice site regions are composed of four nucleotides: A (Adenine), G (Guanine), C (Cytosine), and T (Thymine). Given constraints on the input of DL architectures, these nucleotides are encoded numerically, with each nucleotide corresponding to a row in a 4 × 4 identity matrix. The encoding scheme utilized in this paper is that A corresponds to [1, 0, 0, 0], G corresponds to [0, 0, 1, 0], C corresponds to [0, 1, 0, 0], and T corresponds to [0, 0, 0, 1]. Now consider a family.

$$D = \left\{ {S_{0} , S_{1} , \ldots , S_{n} } \right\}$$

of nucleotide splice site regions. We have the ordered set.

$$S_{i} = \left\{ {x_{1} , x_{2} , \ldots , x_{{\left| {S_{i} } \right|}} } \right\}$$

which $S_{i}$ is the *i*-th nucleotide splice site region, and

$$x_{j} \in X = \left\{ {A, C, G, T} \right\}, 0 \le j \le \left| {S_{i} } \right|$$

For all $0 \le j \le \left| {S_{i} } \right|$ is encoded as a $\left| {S_{i} } \right|*\left| X \right|$ binary matrix through one-hot encoding.

Alternatively stated, if each splice site region consists of some $N$ nucleotides, the final numerical representation for each splice site region is a $N \times 4$ matrix, where each row is a one-hot encoded nucleotide that occurs at the same index as it did in the splice site region's original representation.

We used an easily optimizable hyperparameter tool called KerasTurner (https://keras.io/api/keras_tuner/tuners/hyperband/) for our hyperparameter search. We configure this tool based on the search space parameters as shown in Table 1. This table shows the hyper-parameters, search range, steps, and selected parameters for each CNN and DNN subgroup. To reduce the learning rate as the training proceeds, we used the TensorFlow inverse time decay schedule. For the CNNs, the parameters are initial learning rate 0.001, decay steps 140, and decay rate 0.1, while for the DNNs, the parameters are initial learning rate 0.002, decay steps 80, and decay rate 1.4. We have used a 32-batch size for each neural network model compilation and a 30-epoch for each.

**Table 1:** EnsembleSplice neural network hyper-parameter search space

Neural Network	Hyper-parameter	Range	Steps	Selected
CNN	Filters	8–400	8	72, 120, 136, 144, 168, 208, 250, 272,
	Kernel size	1–9	2	3, 4, 5, 7, 9
	Dropout	0.05–0.30	0.05	0.20, 0.35
	Max-Pool size	1–9	2	3
DNN	Units	32–704	32	32, 128, 224, 250, 256, 352, 512, 704,
	Kernel regularizers	0.0025, 0.025, 0.036	-	0.0025, 0.025, 0.036
	Dropout	0.05–0.50	0.50	0.1, 0.15, 0.25

This table shows the convolutional neural network (CNN) and Dense Neural Network (DNN) search space. This includes the search range, steps and the selected hyperparameter

### Deep learning

Deep learning is a branch of machine learning that uses layered learning and a hierarchical learning model to enable computers to learn complex concepts [26]. Deep Learning is based on an artificial neural network that mimics the concept of brain neurons. Artificial neural networks contain neuronal connections and the ability to send inputs within layers of neurons [26]. Moreso, an artificial neural network with convolutional blocks as its fundamental layers is known as a convolutional neural network. [27–29]. The EnsembleSplice model combines convolutional layers and dense layers networks to receive input, transform it, and output the transformed results between layers to a simple logistics regression. In other words, they combine features and pattern extraction on the genomic acceptor and donor datasets with organized (element-wise multiplication) operations between the layer inputs and their corresponding weights. To detect these patterns, the number and size of filters are given. These filters are matrices with randomly defined values in the rows and columns, allowing for effective differentiation of true/false acceptors and donor splice sites. We tested and analyzed mean cross-validation results for the different ensemble architectures across the acceptor and donor organism datasets to find the best performing model for predicting splice locations. The architecture and model parameters are covered in detail in the EnsembleSplice pipeline section below.

### EnsembleSplice pipeline

**Fig. 2:**
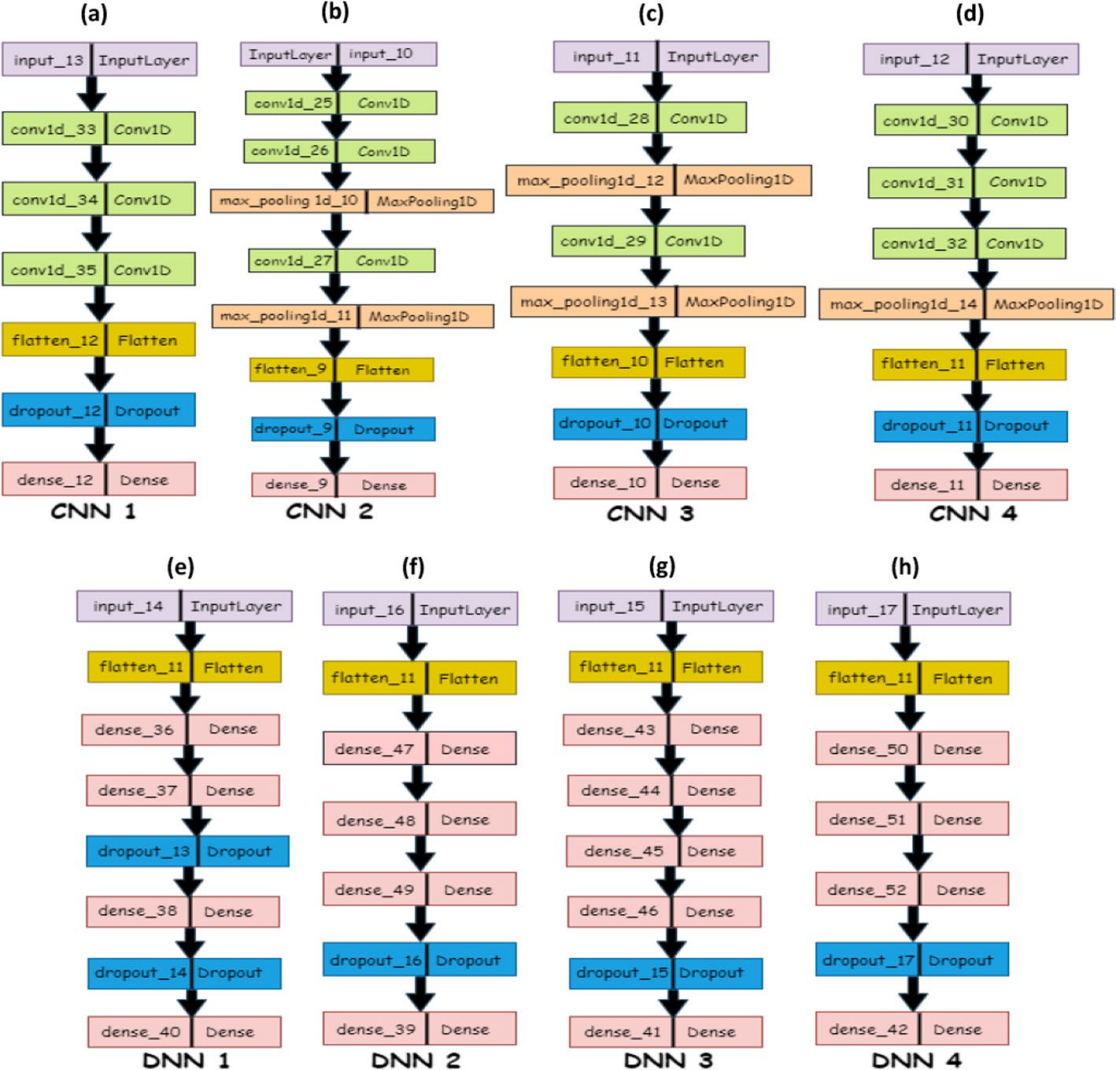
EnsembleSplices’ CNNs and DNNs model architecture. This figure depicts each CNNs and DNNs base model’s architecture used in this cross-validation experiment. This Figure contains **a** CNN 1; **b** CNN 2; **c** CNN 3; **d** CNN 4; **e** DNN 1; **f** DNN 2; **g** DNN 3; **h** DNN 4, with architecture containing its respective layers and their distinct labels

EnsembleSplice is an ensemble learning architecture made up of eight sub-models: four deep neural networks and four convolutional neural networks. The architecture of each CNN and DNN sub-models is shown in Table 2 with colored pattern representation in Fig. 2. Each sub-model's architectural design choices differ significantly. These EnsembleSplice sub-models predict whether inputted genomic DNA sequences are true or false splice regions and handle DoSS and AcSS separately, implying that there are two sets of weights, one for DoSS and the other for AcSS classification. Both DoSS and AcSS use the same sub-model architecture (the architecture of the *i-th* CNN is identical in both). The sub-models produce binary predictions, which are then aggregated (stacked) into a new dataset, with row *i* containing all sub-model predictions for data entry *i*, and this dataset is then fed into an output predictor (logistic regression), which produces a final set of predictions for the inputted nucleotide sequences. Each CNN sub-model in EnsembleSplice is composed of some combination of convolutional layers, a dropout layer, and max-pooling layers. The convolutional layers automatically extract local and global features from the AcSS or DoSS input sequences. These layers create complex representations of the AcSS or DoSS, allowing CNN to distinguish between true and false AcSS/DoSS with accuracy. Each convolutional layer employs the ReLU activation function as its final component; this removes noisy or otherwise irrelevant features, thus improving feature selection [30, 31]. The dropout layer prunes a percentage of each network's total convolutional nodes, which reduces model overfitting by limiting the co-dependencies each node in the network has on other nodes in the network [32]. Each CNN optimizer uses the ADAM optimizer [33] with an inverse time decay learning rate schedule during model compilation.

**Table 2:** EnsembleSplices’ CNNs and DNNs model architecture

Neural networks	Layer type
CNN 1	Conv1D(72, 5)
	Conv1D(144, 7)
	Conv1D(168, 7)
	Flatten()
	Dropout(0.20)
	Dense(2, "sigmoid")
CNN 2	Conv1D(136, 3)
	Conv1D(72, 4)
	MaxPooling1D(7)
	Conv1D(272, 7)
	MaxPooling1D(3)
	Flatten()
	Dropout(rate = 0.35)
	Dense(2, "sigmoid")
CNN 3	Conv1D(208, 9)
	MaxPooling1D(6)
	Conv1D(120, 5)
	MaxPooling1D(3)
	Flatten()
	Dropout(0.20)
	Dense(2, "sigmoid")
CNN 4	Conv1D(250, 5)
	Conv1D(250, 5)
	Conv1D(250, 5)
	MaxPooling1D(3)
	Flatten()
	Dropout(0.20)
	Dense(2, "sigmoid")
DNN 1	Flatten()
	Dense(704)
	Dense(224)
	Dropout(0.1)
	Dense(512)
	Dropout(0.15)
	Dense(2, "sigmoid")
DNN 2	Flatten()
	Dense(704)
	Dense(224)
	Dense(128)
	Dropout(0.15)
	Dense(2, "sigmoid")
DNN 3	Flatten()
	Dense(256)
	Dense(352)
	Dense(32)
	Dense(352)
	Dropout(0.15)
	Dense(2, "sigmoid")
DNN 4	Flatten()
	Dense(250)
	Dense(250)
	Dense(250)
	Dropout(0.25)
	Dense(2, "sigmoid")

The number of filters and kernel size are the first and second parameters for convolutional layers (CNN), respectively, with the same activation function (ReLu) and padding. The pool size is the parameter in the max-pooling layer, and the number of dense nodes and ReLu activation function is the parameter in the layer for dense neural networks (DNNs). DNN 4 uses the random normal as its kernel initializer

Each DNN sub-model in EnsembleSplice consists of several fully connected dense layers, up to 2 dropout layers, and, in some cases, an L2 kernel regularization penalty. Similar to the CNN sub-models, the ReLU activation function and the ADAM optimizer with an inverse time decay learning rate schedule are used.

EnsembleSplice is implemented via the TensorFlow/Keras framework [34, 35]. For all experiments conducted, we use a training maximum epoch of 30. The training and validation were performed in Google Coolaboratory using Graphical Processing Unit (GPU) hardware, and the early model stopping callback, which stops training if the model's validation loss does not decrease for a predetermined number of epochs. The CNN and DNN ensemble sub-model architecture evaluation and selection is discussed in details in the cross-validation section of the Results and Discission section.

### Evaluation metrics

The counts of correctly identified True AcSS or DoSS (true positive, "TP"), correctly identified False AcSS or DoSS (true negative, "TN"), incorrectly annotated True AcSS or DoSS (false positive, "FP"), and incorrectly annotated False AcSS or DoSS (false negative, "FN") are used to evaluate EnsembleSplice's classification performance and to compare EnsembleSplice with other splice site detection models used.

For this experiment, we used evaluation metrics standard to splice site detection research. This includes.

- Accuracy (Acc): the value of AcSS and DoSS correctly identified, given by $Accuracy = \frac{TP + TN}{{TP + TN + FP + FN}}$.
- Precision (Pre): the fraction of positive classifications for AcSS or DoSS that were positive, given by $Precision = \frac{TP}{{{\text{TP}} + FP}}$.
- Sensitivity (Sn): the fraction of positive AcSS or DoSS with a positive classification (true positive rate), given by $Sensitivity = \frac{TP}{{{\text{TP}} + {\text{FN}}}}$.
- Specificity (Sp): the fraction of negative AcSS or DoSS with a negative classification (true negative rate), given by $Specificity = \frac{TN}{{{\text{TN}} + {\text{FP}}}}$.
- Matthew’s Correlation Coefficient (MCC): the correlation between true and false AcSS and DoSS and the classifications for them generated by the mode, given by $MCC = \frac{{{\text{TP}} \times {\text{TN}} - {\text{FP}} \times {\text{FN}}}}{{\sqrt {\left( {{\text{TP}} + {\text{FP}}} \right)\left( {{\text{TP}} + {\text{FN}}} \right)\left( {{\text{TN}} + {\text{FP}}} \right)\left( {{\text{TN}} + {\text{FN}}} \right)} }}$.
- F1 Score (F1): the harmonic mean of the fraction of positive classifications for AcSS or DoSS that were positive and the fraction of positive AcSS or DoSS that were correctly identified, given by $F1 Score = \frac{{2 \times {\text{TP}}}}{{2 \times {\text{TP}} + {\text{FP}} + {\text{FN}}}}$.
- Error Rate: the fraction of AcSS or DoSS incorrectly identified, given by 1 − Accuracy.

We utilized four diversity metrics described below to evaluate how well the different ensembles might generalize in our ensemble cross-validation experiments. They are as follows: correlation, double fault, disagreement, and Q-statistic. For a mathematical illustration of these diversity metrics, we use two classifiers and define $K^{ij}$ as the number of measures for which binary vector $s_{y, x} = i$ and $s_{y, z} = j$. Thus, $K^{11}$ is the number of examples that is correctly classified by the ensemble classifier [36].

Given the output of two classifiers, $Q_{x}$ and $Q_{Z}$:

- Correlation: the correlation is given by $\frac{{K^{11} K^{00} - K^{01} K^{10} }}{{\sqrt {\left( {K^{11} + K^{10} } \right)\left( {K^{01} + K^{00} } \right)\left( {K^{11} + K^{01} } \right)\left( {K^{10} + K^{00} } \right)} }}$. The correlation measure is diverse when the value is low
- Double Fault: this measure the fraction of the misclassified examples by both classifier ensemble and is given by $\frac{{K^{00} }}{{K^{11} + K^{10} + K^{01} + K^{00} }}$. This metric is diverse when the value is low.
- Disagreement: the fraction between the true classifier and false classifier to the total number of examples and is given by $\frac{{K^{01} + K^{10} }}{{K^{11} + K^{10} + K^{01} + K^{00} }}$. Disagreement measure is diverse when the value is high.
- Q-statistics: this measure is given by $\frac{{K^{11} K^{00} - K^{01} K^{10} }}{{K^{11} K^{00} - K^{01} K^{10} }}$. A low value shows high diversity for the Q - statistics metrics.

### Performance benchmark methods

In this study, we chose existing cutting-edge splice site models iSS-CNN [17], SpliceRover [12], SpliceFinder [13] and DeepSplicer [14] for benchmark comparison with EnsembleSplice based on their training architecture, experiment datasets and recent deep-learning based splice site state-of-the-arts.

### ***Tayara ***et al***. ***[17]

iSS-CNN [17], which was trained on a subset of *HS3D* data, has three layers: a dropout layer that prunes 30% of the nodes, a fully connected dense layer using the Sigmoid activation function, one convolutional layer of 16 filters and kernel size 7, stride size 3, and a classification threshold of 0.5 for predicting AcSS or DoSS. The testing was done on the public web server of iSS-CNN and is accessible at http://nsclbio.jbnu.ac.kr/tools/iSS-CNN/. For evaluation, EnsembleSplice uses the same *HS3D* testing subset as the benchmarked iSS-CNN.

### ***Zuallaert ***et al***. ***[12]

SpliceRover [12] which is also a deep learning approach to splice site prediction was trained on human genomic DNA data and *A. thaliana* genomic DNA data. Its architecture consists of a convolutional layer with filters equal in number to the AcSS or DoSS length, a max-pooling layer, and a series of convolutional and max-pooling layers. A fully connected dense layer follows the convolutional layers, and the output is input to the Softmax activation function. When comparing SpliceRover to EnsembleSplice, their publicly accessible web server is used. This time, a cut of 0.5 was used. The web server can be found at the following link: http://bioit2.irc.ugent.be/rover/splicerover.

### ***Wang ***et al***. ***[13]

SpliceFinder [13] was tested on other species of datasets after being trained on the human dataset. Its classification accuracy was 90.25% and it used one-hot encoding, one convolutional layer, a fully connected layer, and Softmax. We use this method as a benchmark for evaluation comparison since it is a more recent splice site prediction method that has been published.

### ***Akpokiro ***et al***. ***[14]

DeepSplicer [14] uses a five-fold cross-validation approach for its model selection. This convolutional neural network state-of-the-art method uses three convolution neural network layers with flatten, dense, dropout, and Softmax layers in its architecture. Similar to EnsembleSplice, this method is trained and tested on *Homo sapiens* and *A. thaliana*. The models, software architecture, and datasets for SpliceFinder [13] and DeepSplicer [14] are all available in the corresponding GitHub repositories.

## Results and discussion

### Cross-validation

To establish a more efficient and consistent model, we performed a five-fold cross-validation experiment. Through this experiment, we estimated the splice site prediction accuracy by dividing the balanced training datasets into K equal dataset splits. This split has an equal number of true and false genomic sequences, with true and false splice sites being genomic sequence patterns with consensus AcSS AG and DoSS GT dinucleotide molecules annotated as splice sites and not annotated as splice sites, respectively. We essentially used the K-1 fold for training and the one-fold for testing for each subset of the data partitions. Finally, the reported accuracy represents the mean accuracy computed from all K data splits across each balanced genomic organism dataset. EnsembleSplice employs the StratifiedKFold [17] ML module for its k-fold (k = 5) cross-validation for each acceptor and donor organism dataset. Consequently, there were five groups from the training datasets.

We tested potential ensemble architectures using the cross-validation method on the following set:

- Ensemble ENS1 contains all DNN’s (DNN1, DNN2, DNN3, DNN4).
- Ensemble ENS2 contains all the CNNs (CNN1, CNN2, CNN3, CNN4).
- Ensemble ENS3 contains all the neural network models (DNN1, DNN2, DNN3, DNN4, CNN1, CNN2, CNN3, CNN4).
- Ensemble ENS4 consists of CNN1, CNN2, CNN3, DNN1, DNN3, this does not include the two worst DNNs and one worst CNN.
- Ensemble ENS5 contains all the neural network sub models except the single worst CNN and DNN (DNN1, DNN3, DNN4, CNN1, CNN2, CNN3).
- Ensemble ENS6 includes all DNNs with the worst DNN removed and all CNNs with the worst two CNNs removed (DNN1, DNN3, DNN4, CNN1, CNN2).

**Fig. 3:**
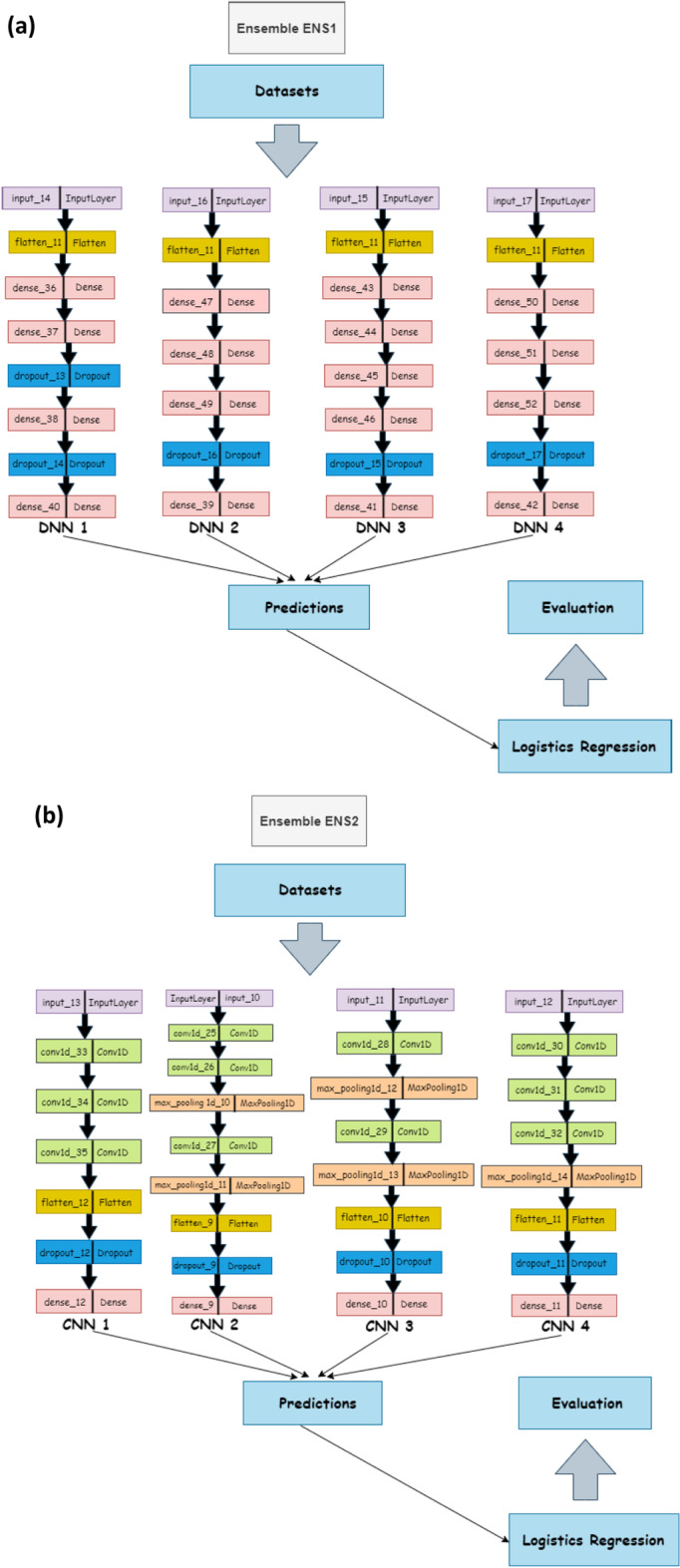

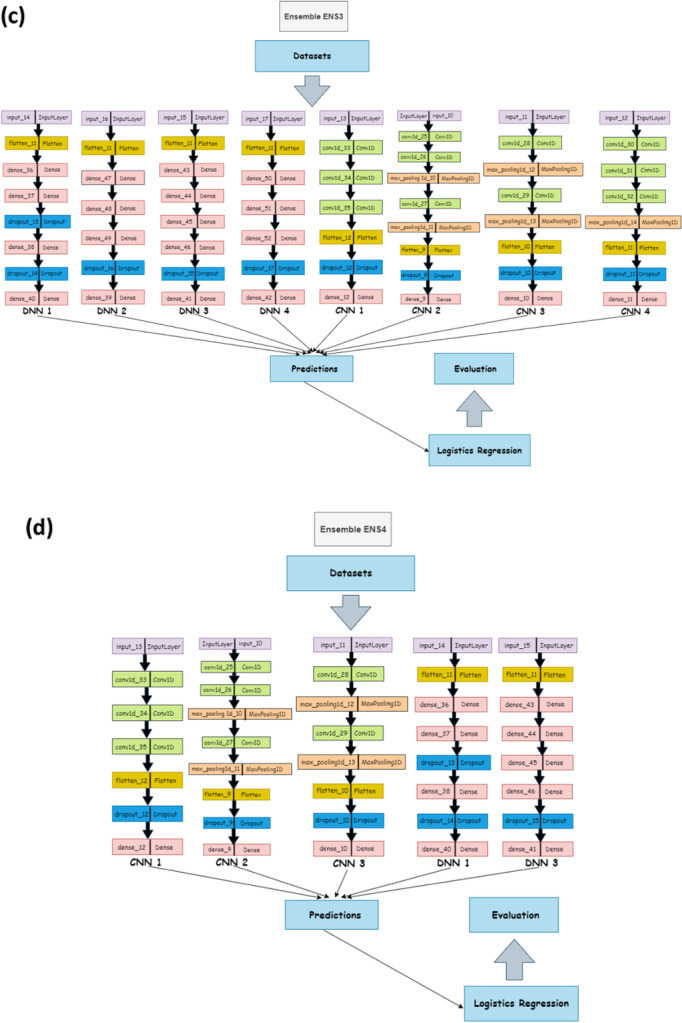

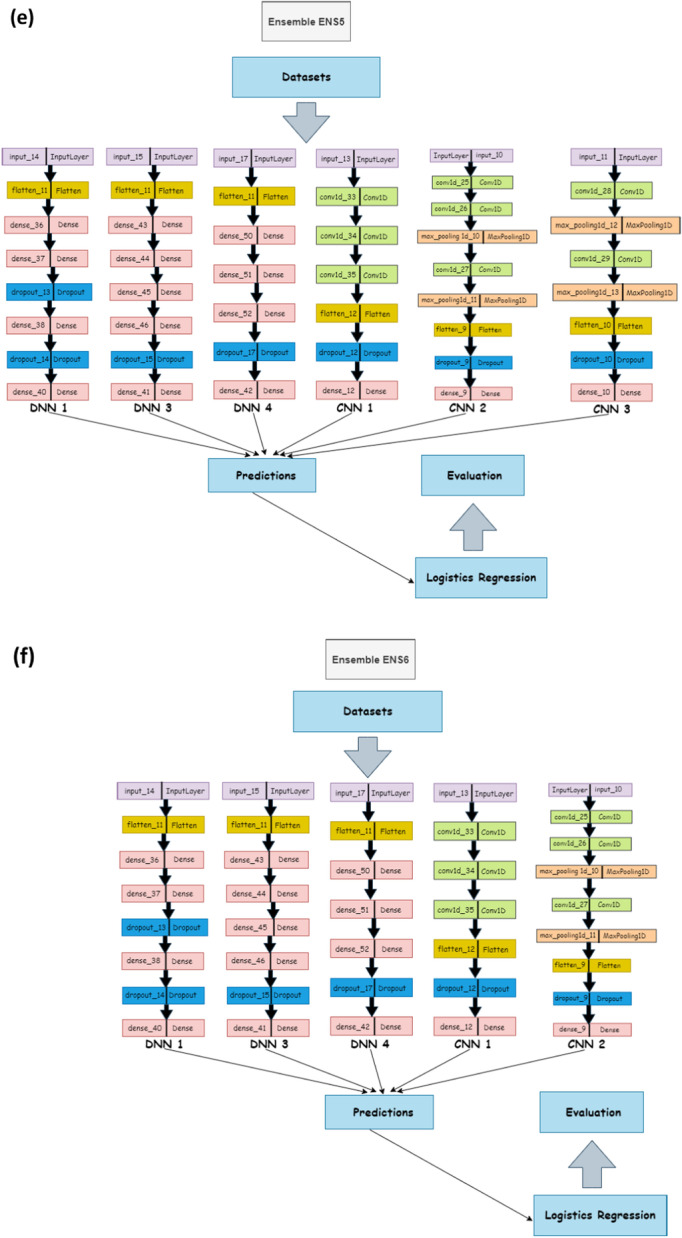
Cross-Validation Ensemble model architecture. These are the architectural representation of each Ensemble model architecture and their individual base model architecture combination used in the cross-validation experiment. This contain **a** Ensemble ENS1 contains all DNN’s (DNN1, DNN2, DNN3, DNN4); **b** Ensemble ENS2 contains all the CNNs (CNN1, CNN2, CNN3, CNN4); **c** Ensemble ENS3 contains all the neural network models (DNN1, DNN2, DNN3, DNN4, CNN1, CNN2, CNN3, CNN4); **d** Ensemble ENS4 consists of CNN1, CNN2, CNN3, DNN1, DNN3; **e** Ensemble ENS5 consists of DNN1, DNN3, DNN4, CNN1, CNN2, CNN3; **f** Ensemble ENS6 consist of DNN1, DNN3, DNN4, CNN1, CNN2. We selected the Ensemble ENS2 from our cross-validation experiment

**Fig. 4:**
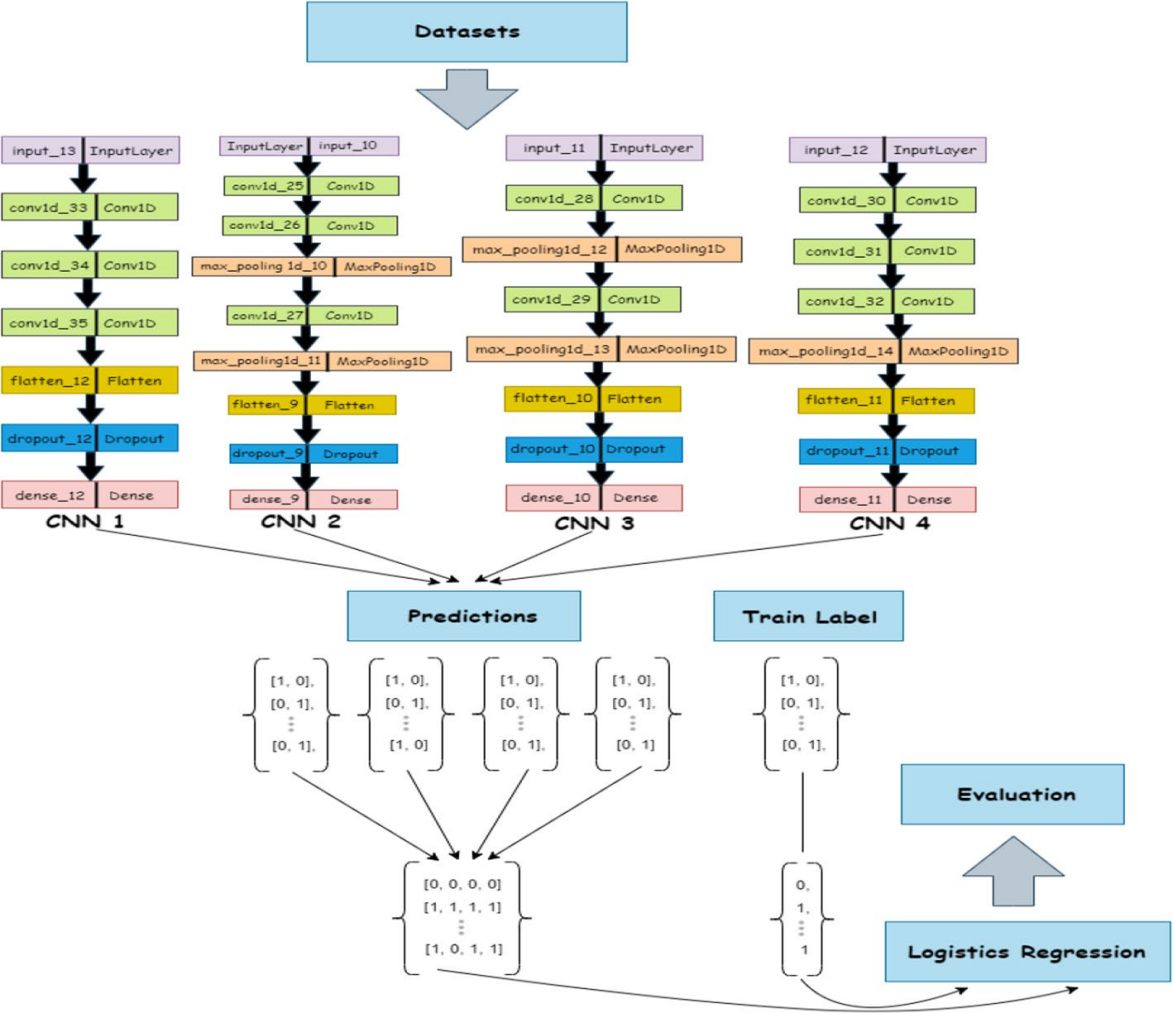
EnsembleSplice architectural pipeline. This figure depicts the Ensemble architecture used for this experiment. This contains the one-hot encoded datasets, the ensemble neural network combination, prediction and label, and the logistics regression and evaluation

The architecture of each of this ensemble sub-models—that is CNNs and DNNs— are provided in Table 2, with the architecture representation in Fig. 3. All the architectures use the one-hot encoding of genome data as their input. Additionally, the output of this architecture serves as the input for a dense and dropout layer. Consequently, we compute the mean results for the evaluation and diversity metrics of the cross-validation results across the organism for each acceptor and donor dataset with results shown in the Table 3. From the table, we observe that the performance of the ENS2 architecture is highly competitive across all the diversity metrics. Importantly, this architecture outperformed the competition in accuracy metrics and error rates for the acceptor and donor splice site datasets for the benchmark organisms. Thus, we selected the ENS2 as the representative EnsembleSplice model. The evaluation metrics section explains the metrics used in this experiment and the Fig. 4 outlines the entire architecture of the ENS2 model, from input, one-hot encoding of the genome data, to output specifying the false and true AcSS/DoSS splice site prediction score.

**Table 3:** The cross-validation results for the dataset for the genomic organisms

Datasets	SpliceSites	Metrics	ENS1	ENS2	ENS3	ENS4	ENS5	ENS6
*HS3D*	Acceptor	Double fault	0.033	0.00	0.01	0.01	0.007	0.011
		Correlation	0.612	0.06	0.22	0.20	0.21	0.33
		Q-statistics	0.89	0.131	0.50	0.65	0.553	0.83
		Disagreement	0.03	0.00	0.03	0.03	0.02	0.03
		Accuracy	0.89	0.936	0.94	0.93	0.94	0.93
	Donor	Double fault	0.013	0.00	0.00	0.00	0.003	0.003
		Correlation	0.496	0.02	0.18	0.11	0.19	0.20
		Q-Statistics	0.796	− 0.001	0.44	0.37	0.451	0.478
		Disagreement	0.015	0.00	0.01	0.01	0.01	0.01
		Accuracy	0.93	0.958	0.95	0.95	0.94	0.94
*A. thaliana*	Acceptor	Double fault	0.023	0.003	0.012	0.01	0.011	0.01
		Correlation	0.667	0.215	0.358	0.401	0.413	0.415
		Q-Statistics	0.988	0.713	0.843	0.98	0.982	0.985
		Disagreement	0.023	0.016	0.097	0.027	0.03	0.025
		Accuracy	0.913	0.947	0.946	0.945	0.948	0.942
	Donor	Double fault	0.013	0.019	0.008	0.006	0.007	0.007
		Correlation	0.638	0.132	0.317	0.3	0.315	0.326
		Q-Statistics	0.992	0.308	0.689	0.83	0.882	0.747
		Disagreement	0.016	0.079	0.089	0.056	0.085	0.016
		Accuracy	0.93	0.954	0.954	0.95	0.953	0.952
*Homo Sapiens*	Acceptor	Double fault	0.034	0.003	0.015	0.01	0.013	0.015
		Correlation	0.702	0.19	0.325	0.338	0.353	0.399
		Q-Statistics	0.989	0.555	0.667	0.978	0.844	0.978
		Disagreement	0.028	0.022	0.083	0.037	0.069	0.037
		Accuracy	0.894	0.938	0.938	0.939	0.937	0.933
	Donor	Double fault	0.022	0.001	0.008	0.007	0.01	0.008
		Correlation	0.665	0.103	0.289	0.298	0.338	0.315
		Q-Statistics	0.989	0.274	0.773	0.894	0.978	0.907
		Disagreement	0.022	0.057	0.024	0.025	0.033	0.025
		Accuracy	0.907	0.952	0.952	0.951	0.949	0.946
Average	Acceptor	Double fault	0.03	**0.002**	0.01	0.02	0.01	0.012
		Correlation	0.66	**0.16**	0.30	0.31	0.32	0.38
		Q-Statistics	0.955	**0.466**	0.58	0.87	0.793	0.931
		Disagreement	0.027	0.012	**0.070**	0.033	0.040	0.030
		Accuracy	0.830	**0.941**	0.940	0.940	0.940	0.930
	Donor	Double fault	0.015	0.012	0.010	**0.004**	0.006	0.008
		Correlation	0.599	**0.09**	0.260	0.240	0.28	0.28
		Q-Statistics	0.9256	**0.193**	0.630	0.700	0.770	0.710
		Disagreement	0.017	**0.045**	0.040	0.030	0.040	0.020
		Accuracy	0.920	**0.954**	0.950	0.950	0.950	0.950

This table depicts the five-fold Cross-validation Results, average result across the organism distribution, evaluation metrics and the ensemble combinations considered. Results highlighted in black shows the best average evaluation metrics. *ENS1* consist of DNN1, DNN2, DNN3, DNN4; *ENS2* consists OF CNN1, CNN2, CNN3, CNN4; *ENS3* consists of DNN1, DNN2, DNN3, DNN4, CNN1, CNN2, CNN3, CNN4; *ENS4* consists of CNN1, CNN2, CNN3, DNN1, DNN3; *ENS5* consist of DNN1, DNN3, DNN4, CNN1, CNN2, CNN3; *ENS6* includes the DNN1, DNN3, DNN4, CNN1, CNN2

### Performance evaluation

We evaluated and compared EnsembleSplice performance to the benchmarked methods based on the metrics described above in the evaluation metrics section and the datasets as discussed in the datasets section with the state-of-the-art methods considered because of their deep learning application. EnsembleSplice outperforms all other methods for the *HS3D* acceptor datasets, with the exception of the precision metrics, where DeepSplicer outperformed EnsembleSplice by a factor of 1.05%. Furthermore, our approach outperforms other cutting-edge methodologies and records an accuracy of 93.79% and a reduced error rate of 6.36%. With an improved accuracy of 96.25% and a reduced error rate of 3.81% in the *HS3D* donor datasets, EnsembleSplice outperforms competing methods. We continued to test EnsembleSplice to predict splice sites in the *A. thaliana* genomic dataset and discovered that it performed better than other methods in every metric for both the acceptor and donor genomic dataset organisms. We tested and compared other splice site models on the Homo-sapiens datasets in order to demonstrate EnsembleSplice's consistency in predicting the splice site. In the acceptor and donor datasets, EnsembleSplice records data with higher accuracy and lower error rates than other methods. In the Table 4 result, N/A denoted results for methods of no known datasets model.

**Table 4:** The Evaluation performance comparison results

Datasets	SpliceSites	Model	Sp	Sn	Pre	Err	Acc	MCC	F1
*HS3D*	Acceptor	ISSCNN	87.27	91.81	87.82	10.45	89.55	79.17	81.45
		SpliceRover	N/A	N/A	N/A	N/A	N/A	N/A	N/A
		DeepSplicer	92.55	92.91	**92.57**	7.27	92.73	85.46	92.74
		SpliceFinder	89.09	93.09	89.51	8.90	91.09	82.24	91.26
		EnsembleSplice	**91.09**	**96.18**	91.52	**6.36**	**93.64**	**87.39**	**93.79**
	Donor	ISSCNN	94.36	94.90	94.39	5.35	94.64	89.27	89.84
		SpliceRover	N/A	N/A	N/A	N/A	N/A	N/A	N/A
		DeepSplicer	95.45	94.36	95.40	5.09	94.91	89.82	94.88
		SpliceFinder	94.00	95.09	94.06	5.45	94.54	89.09	94.57
		EnsembleSplice	**94.37**	**98.00**	**94.56**	**3.81**	**96.18**	**92.43**	**96.25**
*A. thaliana*	Acceptor	SpliceRover	88.31	89.25	88.42	11.22	88.78	77.57	88.83
		ISSCNN	N/A	N/A	N/A	N/A	N/A	N/A	N/A
		DeepSplicer	90.00	94.50	90.43	7.75	92.25	84.59	92.40
		SpliceFinder	90.88	92.69	91.04	8.22	91.78	83.58	91.86
		EnsembleSplice	**93.13**	**95.94**	**93.31**	**5.47**	**94.53**	**89.10**	**94.61**
	Donor	SpliceRover	86.88	87.13	86.91	13.00	87.00	74.00	87.02
		ISSCNN	N/A	N/A	N/A	N/A	N/A	N/A	N/A
		DeepSplicer	90.44	**95.06**	90.86	7.25	92.75	85.59	92.91
		SpliceFinder	93.50	91.13	93.34	7.69	92.31	84.65	92.22
		EnsembleSplice	**94.94**	94.38	**94.91**	**5.34**	**94.66**	**89.31**	**94.64**
*Homo Sapiens*	Acceptor	SpliceRover	88.25	93.44	88.83	9.16	90.84	81.80	91.08
		ISSCNN	N/A	N/A	N/A	N/A	N/A	N/A	N/A
		DeepSplicer	90.88	91.19	90.90	8.97	91.03	82.06	91.04
		SpliceFinder	90.75	89.94	90.67	9.66	90.34	80.69	90.3
		EnsembleSplice	**93.31**	**95.00**	**93.42**	**5.84**	**94.16**	**88.33**	**94.20**
	Donor	SpliceRover	85.44	91.13	86.22	11.72	88.28	76.69	88.61
		ISSCNN	N/A	N/A	N/A	N/A	N/A	N/A	N/A
		DeepSplicer	**96.62**	88.00	**96.31**	7.69	92.31	84.94	91.97
		SpliceFinder	93.00	91.25	92.88	7.87	92.13	84.26	92.06
		EnsembleSplice	96.06	**95.88**	96.06	**4.03**	**95.97**	**91.94**	**95.96**

This table shows the EnsembleSplice splice site prediction performance results and its comparison to other methods which includes iSS-CNN [17], SpliceRover [12], SpliceFinder [13], and DeepSplicer [14]. We show the prediction accuracy measures and the error rate amongst other evaluation metrics performance results. Results figures highlighted in black denotes best performance, N/A are results for methods of no known datasets model. For this table, *Sp* denotes specificity, *Sn* denotes sensitivity, *Pre* denotes precision, *Err* error rate, *Acc* accuracy, *MCC* denotes Mathew’s correlation coefficient, and *F1* denotes the F1 score

Based on the results we have observed and reported above, we can conclude that each of our research objectives have been fulfilled. We have successfully developed a deep ensemble model architecture algorithm for splice site prediction (*objective point 1*). EnsembleSplice is the first deep ensemble model architecture algorithm proposed for splice site prediction. Our method records an outstanding performance in comparison to the state-of-the-art methods and across the evaluation metrics, especially in accuracy and error rate, as shown in Table 4**.** This superior performance can be attributed to both the use of individually effective DNN and CNN architectures for splice site prediction and the use five-fold cross-validation to select the best ensemble architecture capable of generalizing for maximum performance and the diversity of our ensemble-based model to provide model performance robustness *(objective point 2).* Comparing our stable and successful model to other state-of-the-art models, Table 4 demonstrates how the use of ensemble learning for splice site prediction out-performs other cutting-edge models (*objective point 3*).

### Impact and benefit of this study

The primary appeal of deep learning for splice site prediction is that it is more accurate than earlier machine learning methods, especially ones that involved manual feature selection. Although deep learning is somewhat more computationally intensive, it is effective for solving complex problems, which has been its second major appeal. EnsembleSplice further benefits biological research involving splice site classification in that its deep ensemble architecture outperforms individual deep learning networks and exceeds state-of-the-art performance in splice site prediction, not just in terms of accuracy, but also in terms of other classification metrics, such as precision and sensitivity, because of the diverse combination of its base models. Additionally, in this study, we adopt the stacked ensemble learning algorithm which has the major advantage of using a variety of effective models to accomplish classification or regression tasks and produce predictions that perform better than any one model in the ensemble. In our benchmarking results, the performance of EnsembleSplice’s all-CNN stacked ensemble model demonstrates the advantages of using an ensemble architecture over a single CNN model for the prediction of splice sites, and this knowledge may be applied and transferred to other domains to address still unsolved complex regression or classification problems.

### EnsembleSplice model interpretability

**Fig. 5:**
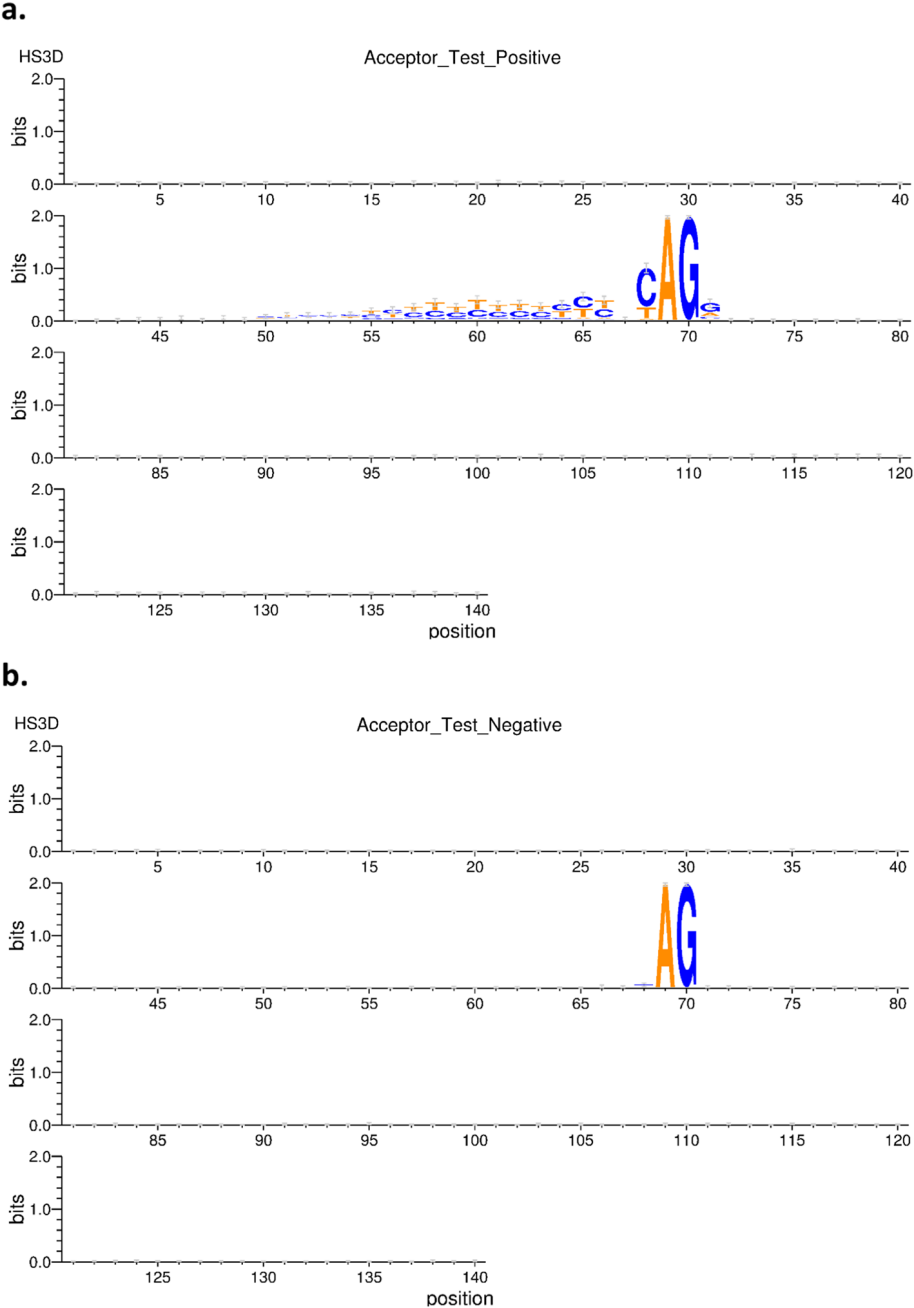

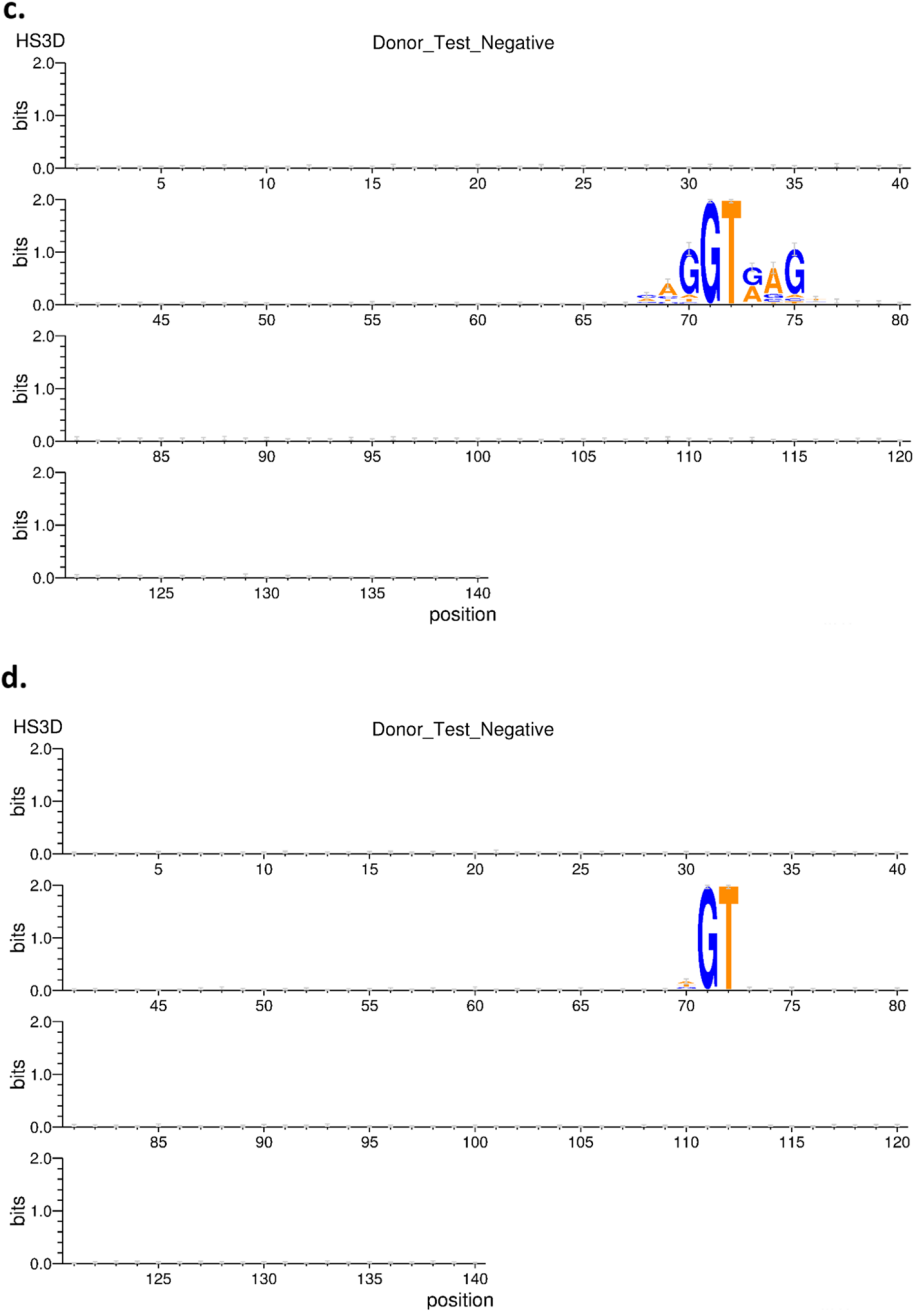
EnsembleSplice model interpretability. This figure is a sequence logo to visualize the importance score for each nucleotide per position for the *HS3D* datasets. **a** indicates the acceptor positive splice sites, as **b** shows that acceptor negative splice sites. While **c** shows the donor positive splice sites as **d** indicates the donor negative splice sites

To increase the model's interpretability, we isolated and showed the motifs that drive our EnsembleSplice model's deep learning processes. Understanding the underlying pattern of the genomic sequence by generating the contribution scores of the sequence window is required for implementation. We used the WebLogo [37] (http://weblogo.threeplusone.com/create.cgi) web server was also used to illustrate the sequence logo for our model interpretability test outputs. WebLogo is a web-based tool for efficiently generating sequence logos from genomic datasets sequence alignment. This genomic sequence logo displays the weighted average nucleotide base position contribution score for the genomic sequence organism. To show the contributions of genomic motifs in each positive and negative acceptor and donor organism dataset, we use the entire *HS3D* sequence length of 140. Figure 5a indicates that the nucleotide sequence AG contributes significantly to the *HS3D* acceptor positive splice sites, as Fig. 5b shows that the nucleotide sequence AG contributes significantly to *HS3D* acceptor negative splice sites. While Fig. 5c shows the nucleotide sequence GT contributes significantly to the *HS3D* donor positive splice sites as Fig. 5d indicates the nucleotide sequence GT contributes significantly to the *HS3D* donor negative splice sites. According to this figure, the nucleotide consensus AG for AcSS regions occurs at positions 69 and 70 and the nucleotide consensus GT for DoSS regions occurs at positions 71 and 72 for the *HS3D* datasets. This figure also validates that the splice site distribution is most significant in sequence region position 70.

## Conclusion

Inspired by the stacking ensemble machine learning method, we introduce a method that combines heterogeneous base neural network models, learns them in parallel, and combines them by training a meta-model to output a prediction based on the different base model predictions. EnsembleSplice has the advantage of balancing out the base model's flaws and produces a diverse and stable model that can be applied to both competitive, industrial, and academic research applications. EnsembleSplice has consistently shown competitive performance on all metrics used when compared to other methods considered in this experiment. As it contributes computationally to the foundation of protein synthesis and gene expression, this tool finds use in both industrial and academic research applications. In our future work, we will test the generalization strength of the EnsembleSplice model for the prediction of splice sites in DNA sequences across a variety of species.

## Data Availability

The datasets, models and codebase for this study are available at https://github.com/OluwadareLab/EnsembleSplice. The Python source codes for EnsembleSplice are available at https://github.com/OluwadareLab/EnsembleSplice.

## Abbreviations

SS: Splice site
CNN: Convolutional neural networks
DL: Deep learning
ReLU: Rectified linear unit
ML: Machine learning
SVM: Support vector machines
MM: Markov model
MDD: Maximum dependency decomposition
AG: Adenine–Guanine
GT: Guanine–Thymine
